# RoseAP: an analytical platform for gene function of *Rosa rugosa*

**DOI:** 10.3389/fpls.2023.1197119

**Published:** 2023-06-30

**Authors:** Lingling Da, Jiande Li, Fan Zhao, Huilin Liu, Pengxia Shi, Shaoming Shi, Xinxin Zhang, Jiaotong Yang, Hui Zhang

**Affiliations:** ^1^ College of Life Science, Northwest Normal University, Lanzhou, China; ^2^ Resource Institute for Chinese and Ethnic Materia Medica, Guizhou University of Traditional Chinese Medicine, Guiyang, China

**Keywords:** *Rosa rugosa*, functional annotation, co-expression network, expression profile, transcriptome

## Abstract

*Rosa rugosa*, a perennial shrub belonging to family Rosaceae, is a well-known ornamental plant. Its petals contain an abundance of essential oils and anthocyanins with enormous economic and health benefits when used as edible or cosmetic ingredients. The whole genome of *R. rugosa* was sequenced in 2021, which provided opportunities and challenges for gene regulation. However, many gene functions remain unknown. Therefore, an analytical platform named RoseAP (http://www.gzybioinformatics.cn/RoseAP/index.php) for the functional analysis of *R. rugosa* genes was constructed. It improved the gene annotation rate by integrating and analyzing genomic and transcriptomic datasets. First, 38,815 genes, covering 97.76% of the coding genes, were annotated functionally and structurally using a variety of algorithms and rules. Second, a total of 33 transcriptome samples were integrated, including 23 samples from our lab and 10 samples from the SRA database. A co-expression network containing approximately 29,657 positive or negative gene pairs, covering 74.7% of the coding genes, was constructed based on PCC and MR algorithms. Network analysis revealed that the DFR function was closely related to anthocyanin metabolism. It demonstrated the reliability of the network. Several *SAUR* genes of *R. rugosa* shared similar expression patterns. RoseAP was used to determine the sequence, structure, functional annotation, expression profile, regulatory network, and functional modules at the transcriptional and protein levels by inputting gene IDs. In addition, auxiliary analytical tools, including BLAST, gene set enrichment, orthologue conversion, gene sequence extraction, gene expression value extraction, and JBrowse, were utilized. Regular updates to RoseAP are expected to facilitate mining of gene function and promote genetic improvement in *R. rugosa*.

## 1 Introduction

*Rosa rugosa*, a perennial shrub belonging to family Rosaceae, is a well-known ornamental species. The essential oil extracted from its petals, known as “liquid gold”, is invaluable as an edible or cosmetic ingredient for its economic and health benefits (Ng et al., 2004; Ren et al., 2018; Zhang et al., 2019a). Nonetheless, its bright and fragrant petals contribute to its ornamental value. Therefore, it has literary and artistic value, and has inspired poets, painters, sculptors, and other artists down the ages. In addition, it is widely cultivated because of its strong adaptability to drought, saline and alkaline conditions, high temperature, and humidity. Thus, it has strong ecological advantage and high breeding value for improving the resistance of other rose plants (Chen et al., 2021; Wang et al., 2021).

The whole genome of *R. rugosa* was sequenced and reported in two versions (Chen et al., 2021; Zang et al., 2021). The whole genome sequencing has accelerated gene regulation and breeding of rose via efficient mining and analysis of complex omics and big data. Currently, transcriptome sequencing has become an important strategy to study gene function (Li and Li, 2018). The expression of genes can be directly reflected compared with the reference genome. Thus, the differentially expressed genes can be screened combined with the phenotype analysis to enhance the identification of phenotype-related functional genes (An et al., 2020; Wei et al., 2020). The accumulation of transcriptomic datasets in several plant and animal species may be of limited value following complete screening of differentially expressed genes by individual researchers. However, with the accumulation of additional data, the mining of omics data is not limited to individual samples. The integration and analysis of a large number of data from different platforms provides new ideas, given the complex molecular networks in cells. The construction of gene co-expression network reflecting the gene expression can be used to explore the relationship between gene expression and function, and thereby facilitate the study of the potential genetic mechanism underlying important agronomic traits (Li et al., 2016; de Los Reyes et al., 2017; Zhang et al., 2019b).

Data integration and reuse has been utilized in several species. Public databases have been developed to facilitate corresponding query analysis, such as MCENet (http://bioinformatics.cau.edu.cn/MCENet/) for maize (Tian et al., 2018), VTCdb (http://vtcdb.adelaide.edu.au/home.aspx) for grape (Wong et al., 2013), WheatCENet (http://bioinformatics.cau.edu.cn/WheatCENet) for wheat (Li et al., 2022), AppleMDO (http://bioinformatics.cau.edu.cn/AppleMDO) for apple (Da et al., 2019), and ATTED-II (http://atted.jp/) for several plants (Obayashi et al., 2022).

The development of various sequencing technologies contributes to the analysis of characteristics of organisms in different dimensions at the whole genome level. In *R. rugosa*, however, the functions of most genes are still unknown, and the results of integrated omics analysis of gene function are still weak. The GDR database (https://www.rosaceae.org) provides genome information and partial annotation information for further studies involving rose (Jung et al., 2019). However, compared with data involving Arabidopsis, rice, and other plants, the *R. rugosa* database is not available publicly, contributing to low rate of data utilization. Therefore, we constructed a database to improve the annotation rate of *R. rugosa* by integrating and analyzing genomic and transcriptomic datasets to facilitate high-quality cultivation of *R. rugosa*.

## 2 Materials and methods

### 2.1 RNA-seq data processing

RNA-seq was performed based on specific concentration and completion criteria (**Supplementary Table 1**). For the raw data of sequencing, the FastQC software (version 0.11.2) (https://www.bioinformatics.babraham.ac.uk/projects/fastqc) was used to assess the quality. The low-quality data were processed using FASTX software (version 0.0.13) (http://hannonlab.cshl.edu/fastx_toolkit) to filter low-quality reads. Cutadapt software (version 1.8.3) (http://cutadapt.readthedocs.io/en/stable) and fastx_trimmer in the FASTX were used to remove adaptor sequences. After processing, FastQC was used again to determine the quality, and if the quality was still too low, the data were discarded.

The qualified data were aligned to the reference genome (http://eplantftp.njau.edu.cn/Rosa_rugosa/) (Chen et al., 2021) using HiSAT2 (version 2.2.1) and Samtools (version 0.1.19). The TPM (transcripts per kilobase of exon model per million mapped reads) values of each gene in each sample were extracted from the Bam file using StringTie (version 2.2.0) (Pertea et al., 2015), according to the gtf annotation file. The TPM was calculated as follows:


$$\text{TPM}=\frac{\frac{N_{i}}{L_{i}}*10^{6}}{Sum\left(\frac{N_{1}}{L_{1}}+\frac{N_{2}}{L_{2}}+\ldots +\frac{N_{n}}{L_{n}}\right)\;}$$

where *N* represents the number of reads over a mapped exon and *L* represents the length of the gene.

### 2.2 Construction of gene co-expression network

Cluster analysis of samples was performed to eliminate outliers using the pheatmap package in R language (version 1.0.8), based on the TPM values of all genes in different samples. Next, Pearson correlation coefficient (PCC) and Mutual Rank (MR) were used to measure the similarity of expression between genes. The PCC algorithm was used to calculate the similarity of variation in the expression of the two genes in samples using the adjacency function of the WGCNA package in R language. MR algorithm was used to calculate the rank of PCC values using the rank function in R language with the parameter ties.method as “min”. The formulas used to calculate PCC and MR are as follows:


$$\text{PCC}=\frac{\sum^{}\left(X-\bar{X}\;\right)\;\left(Y-\bar{Y}\;\right)\;}{\sqrt{\sum_{i=1}^{n}\;\left(X_{i}-\bar{X}\right){\;}^{2}}\;\sqrt{\sum_{i=1}^{n}\;\left(Y_{i}-\bar{Y}\right){\;}^{2}}\;}$$

$$\text{MR }\left(\text{AB}\right)=\sqrt{Rank\;\left(A\to B\right)\times Rank\;\left(B\to A\right)}$$

In the formulas above, n is the sample number of all RNA-seq data, and **x** and **y** denote TPM values. Rank refers to the ranking of PCC values, whereas A→B represents the ranking of gene A in all genes with gene B, and B→A indicates the opposite.

Furthermore, the network reliability was evaluated, and the threshold values for the PCC and MR of the network were set. The GO terms for biological processes with a number of genes ranging from 4 to 20 were selected as prior gene sets. The co-expressed genes under the threshold were selected as the other gene sets. The areas under the ROC curve (AUC) under thresholds were compared. The PCC and MR with the maximum AUC, that is, the maximum crossover of two type gene sets, were selected as the network threshold. In addition, the top three gene pairs with PCC values of each gene were also reserved.

### 2.3 Module identification

Closely related gene groups in the network, that is, co-expression modules, were identified using the CFinder software (version 2.0.6) based on the clique percolation method (CPM). The parameter k = 6 was set to limit the co-expression of each gene and at least six genes in a module (Derenyi et al., 2005; Adamcsek et al., 2006). Furthermore, we annotated the function of these modules via gene set enrichment analysis based on PlantGSAD (http://systemsbiology.cau.edu.cn/PlantGSEAv2/) (Ma et al., 2021).

### 2.4 Gene family identification

The families of transcription factor and protein kinase were identified using the iTAK software (http://bioinfo.bti.cornell.edu/cgi-bin/itak/index.cgi) (Jin et al., 2017). Each family was screened according to the E-value assigned by the UUCD database (http://uucd.biocuckoo.org/) (Gao et al., 2013). Unique domains of transcription factors need to be considered. For example, the F-box family members contain PF00646 and PF12937 domains, and the RING family members carry PF13639 domains. The ubiquitin family was identified using the local version of the HMM model in the UUCD database under default parameters. The carbohydrate-active enzymes, cytochrome p450, epigenetic regulators, and transporters were predicted based on orthologue pairs in *Arabidopsis thaliana*, which were collected from the CAZy database (http://www.cazy.org/) (Lombard et al., 2014), Cytochrome P450 database (http://drnelson.uthsc.edu/CytochromeP450.html) (Nelson, 2009), ChromDB database (http://www.chromdb.org/) (Gendler et al., 2008), and TransportDB database (http://www.membranetransport.org/transportDB2/index.html) (Elbourne et al., 2017), respectively.

### 2.5 Functional annotations

The Gene Ontology (GO) annotations were predicted by aligning protein sequences with the ClusteredNR database of NCBI (https://www.ncbi.nlm.nih.gov) using Blastall. The NR annotations were converted into GO annotations using Blast2GO (https://www.blast2go.com) (Conesa and Gotz, 2008). The Kyoto Encyclopedia of Genes and Genomes (KEGG) metabolic pathways, InterPro, trEMBL, and SwissProt annotations were downloaded from the GDR database (https://www.rosaceae.org) (Jung et al., 2019). Conserved domains were identified using PfamScan (https://www.ebi.ac.uk/Tools/pfa/pfamscan/) based on Multiple Sequence Alignment and HMMER3 (El-Gebali et al., 2019).

### 2.6 Orthologue conversion

Orthologue predictions were performed using InParanoid (version 4.2) (http://inparanoid.sbc.su.se/cgi-bin/index.cgi) (Persson and Sonnhammer, 2022) based on the Blastall program of Blast software (version 2.2.19), with bootstrap ≥ 0.6. The protein sequences of each species were downloaded from public databases as follows: *A. thaliana* from TAIR (https://www.arabidopsis.org); *R. wichuraiana*, *R. multiflora*, and *R. chinensis* from the GDR database (https://www.rosaceae.org); *Malus domestica* from The Apple Genome and Epigenome (https://iris.angers.inra.fr/gddh13); *Paeonia ostia* from the CNGB database (https://ftp.cngb.org/pub/CNSA/data5/CNP0003098/CNS0560369/CNA0050666), and other species (*Prunus persica*, *Fragaria vesca*, *Solanum lycopersicum*, *Populus trichocarpa*, and *Vitis vinifera*) from the Phytozome database (https://phytozome-next.jgi.doe.gov/).

### 2.7 Heatmap analysis

The expression values of all coding genes in all samples were formatted as a matrix, with samples represented horizontally and genes along the vertical axis. The pheatmap program (version 1.0.12) in the R package (https://cran.r-project.org/src/contrib/Archive/pheatmap/) is used for clustering and visual display. The *x*-coordinate samples are fixed without clustering, and the *y*-coordinate genes are clustered. Two types of clustering results can be obtained in RosaAP. First, the TPM values can be directly used for heatmap analysis. Second, the TPM matrix can be standardized, that is, converted to *Z*-scores.


$$Z-\text{score}=\frac{{\text{TPM}}_{\text{i}}-{\text{TPM}}_{\text{mean}}}{{\text{TPM}}_{\text{stdev}}}$$

### 2.8 Other analytical tools

Blast analysis: Blast (version 2.2.19) was installed in the background server. Protein and nucleic acid libraries were established for *R. rugosa* and Arabidopsis with protein and transcript sequences, respectively. Default parameters can be used or set by the user.

Gene set enrichment analysis: Gene set enrichment analysis was used to identify significantly enriched annotation terms for gene sets of interest based on GO annotation, KEGG pathway, and gene family, according to PlantGSAD (http://systemsbiology.cau.edu.cn/PlantGSEAv2/) (Ma et al., 2021).

Orthologue conversion: The predicted orthologues for *R. rugosa* were stored in the MySQL database and directly accessed and downloaded by users in RoseAP.

Expression value extraction: The gene expression profiles were stored in the MySQL database, including TPM and FPKM values for direct access and download by users in RoseAP.

Sequence extraction: Protein and transcript sequences extracted based on gene ID were saved into the MySQL database. Sequence extraction by location was performed using the substr program in Perl (version 5.16.3) (https://www.perl.org).

JBrowse: The alignment results of the transcriptomic datasets and gff3 annotation file were uploaded to the JBrowse to visually display the gene expression profiles and structural features.

## 3 Database contents

### 3.1 Gene functional annotation

The genome sequencing data were downloaded from the eplant database (http://eplantftp.njau.edu.cn/Rosa_rugosa), including the whole genome sequence, gff3 annotation file of genomic features, protein sequence, and transcript sequence. Seven chromosomes, 382.6Mb in size, were annotated to 39,704 coding genes (Chen et al., 2021). Currently, the function of many genes in *R. rugosa* is still unknown. Therefore, the functional annotation of genes is provided in RoseAP. Here, we annotated 38,815 genes, including structural and functional annotation, covering 97.76% of the coding genes.

**Figure 1:**
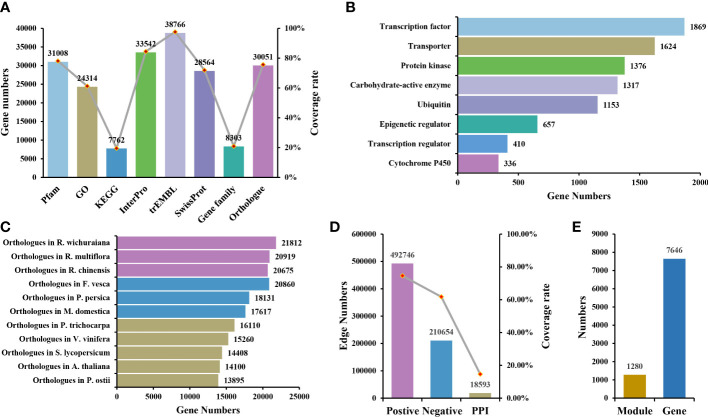
Overview of RoseAP. **(A)** Annotated information of gene structure and function. **(B)** Information of eight gene families identified. **(C)** Orthologous genes of *Rosa rugosa* with 11 other species. **(D)** Number of edges and gene coverage rate in the co-expression and protein–protein interaction network. **(E)** The number of functional modules and genes involved.

As the key region of genes, the domain is closely related to gene function. Genes in the same gene family often contain the same domain. In *R. rugosa*, 31,008 genes were annotated to the conserved domain, covering 78.1% of the coding genes. In addition to structural annotation, many functional annotations were provided, including GO, InterPro, trEMBL, SwissProt and KEGG, covering 61.24%, 84.48%, 97.64%, 71.94%, and 19.55% of the coding genes, respectively, which improved the degree of gene functional annotation in *R. rugosa* (**Figure 1A**).

Eight gene families were identified based on specific algorithms and prior knowledge, covering 8,303 genes, which were divided into 831 gene sets. We classified 1,317 genes in the carbohydrate-active enzyme family (99 sub-families), 336 genes in the cytochrome P450 family (94 sub-families), 657 genes in the epigenetic regulator family (358 sub-families), 1,376 genes in the protein kinase family (92 sub-families), 1,869 genes in the transcription factor family (64 sub-families), 410 genes in the transcription regulator family (25 sub-families), 1,624 genes in the transporter family (76 sub-families), and 1,153 genes in the ubiquitin family (23 sub-families) (**Figure 1B**).

Owing to the evolutionary conservation among species, the functions of homologous genes are also similar to some extent. Therefore, the orthologous genes of related species and model plants are provided as references to enhance our understanding of gene function in *R. rugosa*. Eleven species share orthologous genes with *R. rugosa* in RoseAP, including *A. thaliana*, the best annotated model plant; *Solanum lycopersicum*, a model fruit-bearing crop; *R. wichuraiana*, *R. multiflora*, and *R. chinensis* under the same genus; *Fragaria vesca*, *Prunus persica*, and *Malus domestica* in the Rosaceae family; *Populus trichocarpa* and *Vitis vinifera*, earlier whole-genome sequenced plant species; and *Paeonia ostii*, one of the more ornamental garden plants (**Figure 1C**).

### 3.2 Co-expression network analysis

The 33 samples of transcriptome included five tissue types (flower, stamen, pistil, petal, and leaf), of which 10 samples were derived from the SRA database (SRP167002 and SRP140119) (https://www.ncbi.nlm.nih.gov/sra) in NCBI (Guo et al., 2019). Another 23 samples were generated in our lab based on materials grown in experimental fields, treated without stress, and sampled during the flowering stage. All RNA-seq datasets were aligned to the reference genome, with an overall mapping ratio greater than 60% (**Supplementary Table 2**). The samples were clustered based on TPM value, and an outlier sample was abandoned. As shown in **Supplementary Figure 1**, the final clustering results revealed a single category of leaves, while the related tissues of flower organs were grouped into a large category comprising distinct stamen and pistil components.

Initially, the PCC values of expression profile between genes were calculated to determine the correlation. The larger the absolute value of PCC, the stronger the correlation. When the value of PCC is equal to 1, it suggests completely consistent expression profile of the two genes. Conversely, when the value of PCC is equal to −1, it indicates the completely opposite correlation. Based on the distribution map of PCC values, most gene pairs were not correlated with expression or showed weak correlation (**Supplementary Figure 2A**). Primarily, the top 5% of gene pairs with the highest PCC in the range [0.55, 1] were considered positively correlated, while the last 5% of gene pairs with the lowest PCC value in the range [−1, −0.4] were negatively correlated (**Supplementary Figure 2A**). Accordingly, the co-expressed gene pairs were screened using MR values, which defined the degree of proximity between two genes according to the rank of PCC values in each other’s network. The smaller the MR value, the stronger the correlation. Furthermore, *a priori* gene set was used to evaluate the reliability of the network and select more stringent parameters. Because genes that participate in the same biological activity tend to have similar expression profiles, we selected GO terms (Biological Process) as the prior gene set, in which 405 GO terms with the number of genes ranging between 4 and 20 were selected. The area under curve (AUC) under different PCC and MR values was compared based on the overlap between positive co-expressed genes and the GO gene sets, and the optimal value was selected as the network threshold. The network threshold was set as PCC ≥ 0.8 and MR ≤ 40 for the positive co-expression network (**Supplementary Figure 2B**). In addition, the negative co-expression network also reflected biological activity, and its thresholds were set as PCC ≤ −0.5 and MR ≤ 50, suggesting that the number of gene pairs was nearly half of the number in the positive co-expression network. Finally, this co-expression network for *R. rugosa*, covering 74.70% of the coding genes, carried approximately 700,000 co-expression gene pairs, including positive and negative co-expression networks comprising approximately 490,000 and 210,000 gene pairs, respectively (**Figure 1D**).

Furthermore, 1,280 modules were identified, containing 7,646 genes. These modules may be collectively involved in some biological activity (**Figure 1E**). They were annotated functionally via gene set enrichment analysis. Several modules were related to various biological processes, such as CFinderM1259 related to “response to heat” (http://www.gzybioinformatics.cn/RoseAP/module_detail.php?module=CFinderM1259), CFinderM0239 related to “regulation of flower development” (http://www.gzybioinformatics.cn/RoseAP/module_detail.php?module=CFinderM0239), and CFinderM1224 related to “ion transport” (http://www.gzybioinformatics.cn/RoseAP/module_detail.php?module=CFinderM1224).

### 3.3 Protein–protein interaction network

The study of biological activities with complex regulatory systems based on a single perspective is limited by specific constraints, suggesting the need to consider interactions at the protein level. For *R. rugosa*, we obtained a total of 18,593 pairs of interacting proteins, covering 5,805 (14.62%) genes, via orthologous gene alignment with Arabidopsis PPIs from TAIR (https://www.arabidopsis.org/), BAR (http://bar.utoronto.ca/welcome.htm), and BioGRID (http://thebiogrid.org/) (**Figure 1D**).

### 3.4 Analytical tools

In addition, several support tools are available in RoseAP. The BLAST analysis tool provides sequence alignment for *R. rugosa* and *A. thaliana* by building libraries of protein and CDS sequences for each species, respectively. Gene set enrichment analysis tool provides functional annotation of gene sets based on GO annotation, KEGG pathway, and gene families. The orthologue conversion tool provides queries of orthologous genes with 11 species, including model species and closely related species, based on input of a single gene or multiple genes. The sequence extraction tool can be used to obtain a gene sequence based on its ID or location on chromosomes. The expression value extraction tool provides gene TPM or FPKM value in different tissues that can be searched by gene ID. The JBrowse tool provides a visual display of gene expression profiles for every exon.

**Figure 2:**
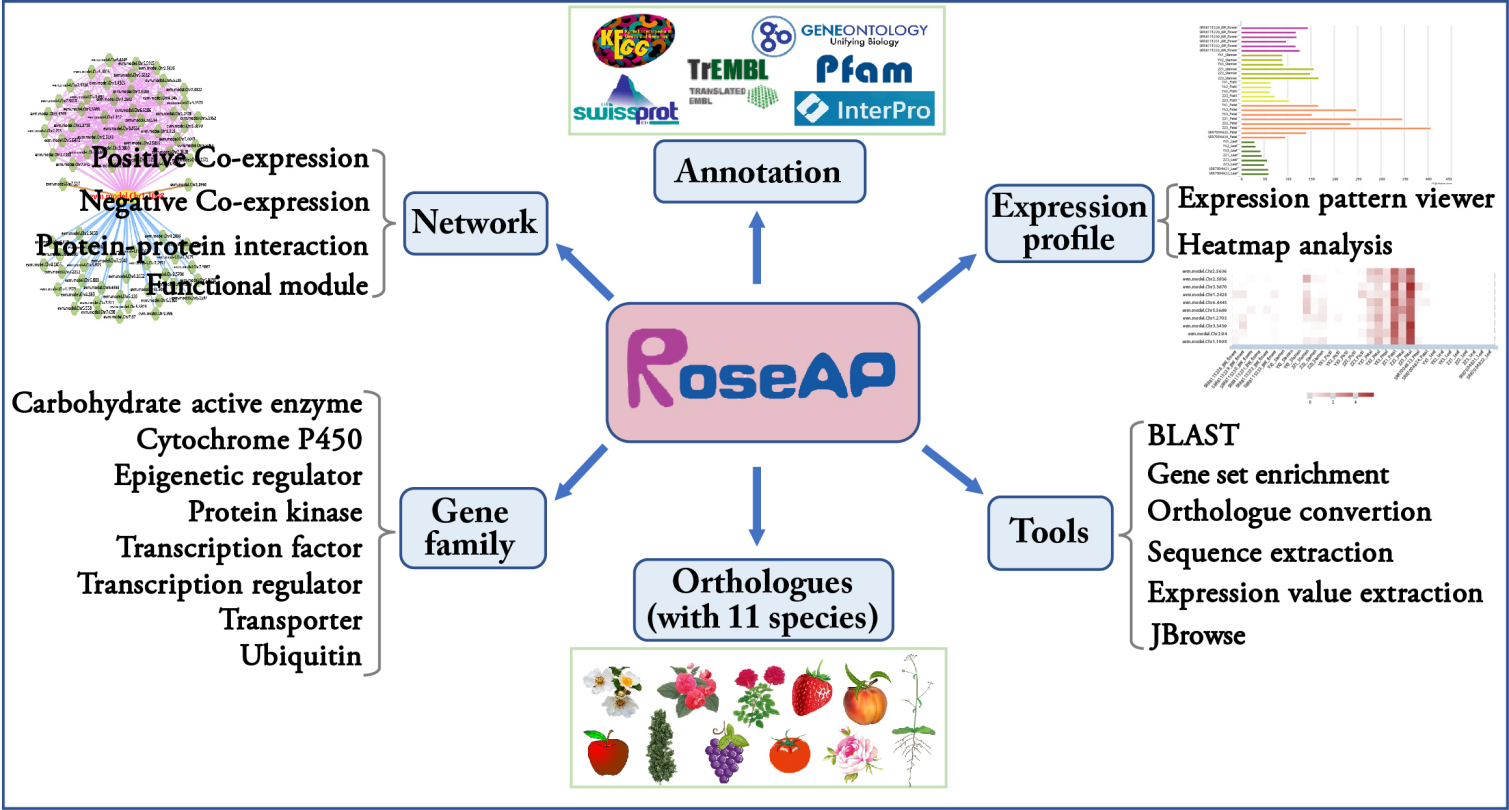
Outline of RoseAP.

Based on the above results, an *R. rugosa* functional analysis platform RoseAP was constructed, including basic annotation, expression profile, network analysis, gene family, orthologue, and six tools (**Figure 2**).

## 4 Case study

### 4.1 Basic functional and structural analysis

**Figure 3:**
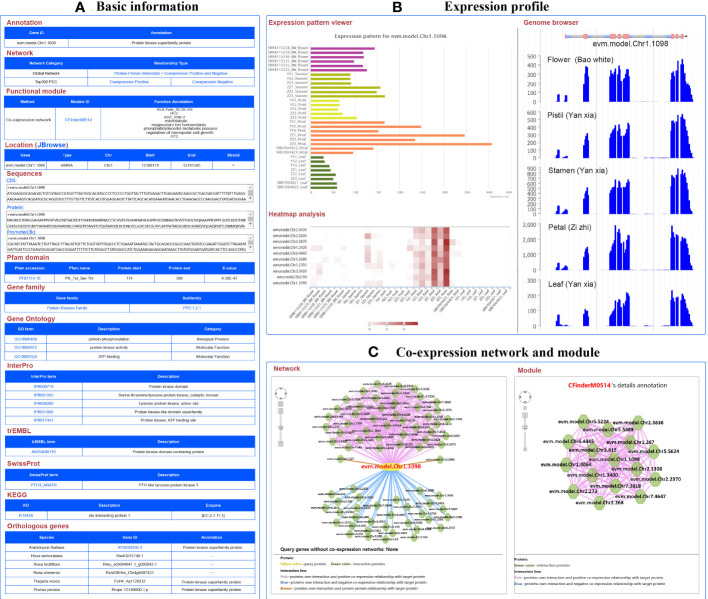
A basic description of single gene analysis in RoseAP. **(A)** Basic analysis of evm.model.Chr1.1098, including annotation, network, functional module, gene location, sequences, pfam domain, gene family, gene ontology, InterPro, trEMBL, SwissProt, KEGG, and orthologous genes for evm.model.Chr1.1098. **(B)** Expression profile analysis of evm.model.Chr1.1098, including expression pattern viewer, genome browser, and heatmap analysis of some genes related to search genes submitted by users. Based on expression pattern viewer, the color of the bar chart is used to distinguish different tissues. For genome browser, peaks represent the distribution of raw reads of RNA-seq datasets across the genome. For heatmap analysis, the redder the color, the higher the activity of the gene. **(C)** Network analysis and module search for evm.model.Chr1.1098. The yellow circle is the search gene, and the polygons represent genes co-expressed or protein–protein interactions with the search gene. The pink, blue, and brown lines indicate positive co-expression, negative co-expression, and protein–protein interaction with the search gene, respectively.

For example, the gene evm.model.Chr1.1098 was identified as a member of the protein kinase family, located on chromosome 1 from 12,186,119 to 12,191,345 bp. Its CDS and protein sequences were also provided. Furthermore, it was found that the PK_Tyr_Ser-Thr domain was located at 114 to 338 bp in the sequence using PfamScan software (https://www.ebi.ac.uk/Tools/pfa/pfamscan/), which was numbered as PF07714.18, which can be used to access the Pfam website (http://pfam-legacy.xfam.org/) (Mistry et al., 2021). This term represents the catalytic domain in a number of serine/threonine- and tyrosine-protein kinases. This gene belongs to receptor-like cytoplasmic kinase VIII subfamily of the protein kinase family. The other members of this subfamily can also be found based on the PPC:1.2.1 link. GO annotation revealed one BP term (protein phosphorylation) and two MF terms (protein kinase activity and ATP binding). KEGG-annotated pto-interacting protein 1 and SwissProt-annotated PTI1-like tyrosine-protein kinase 3 suggest that the gene might code a PTI1-associated protein. In addition, annotations from InterPro and trEMBL also indicated that the gene functions as a protein kinase. The annotated information confirms the function of this gene from multiple perspectives (**Figure 3A**).

The expression profile of this gene was significantly higher in flower than in leaves, and higher in petals than in other parts of the flower. The expression level of this gene was higher in “ZiZhi” than in the “YanXia” variety (**Figure 3B**). Furthermore, we also conducted a heatmap analysis of a few genes to directly compare evm.model.Chr1.1098 with its expression profiles (**Figure 3B**). In addition, network analysis showed 42 positive co-expressed genes with PCC ≥ 0.86, 32 negative co-expressed genes with PCC ≤ −0.65, and 2 protein-interacting genes for evm.model.Chr1.1098 (**Figure 3C**). The color of edge in the network represents the relationship between genes, in which red, blue, and brown represent positive co-expression, negative co-expression, and protein–protein interactions, respectively. The PCC values are listed in the table below the network diagram, with each gene providing annotation information. The module analysis revealed that evm.model.Chr1.1098 was involved in module CFinderM0514, including 16 genes. The function of this module was related to protein kinase and phosphatidylinositol metabolism (**Figure 3C**) (http://www.gzybioinformatics.cn/RoseAP/module_detail.php?module=CFinderM0514).

### 4.2 Co-expression network analysis of small auxin up RNAs

**Figure 4:**
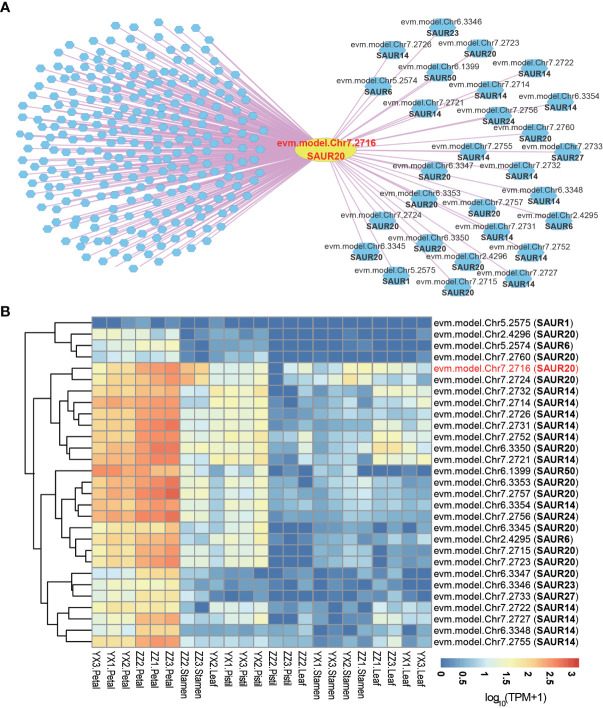
Co-expression network analysis of small auxin up RNAs. **(A)** The network of *SAUR20* (evm.model.Chr7.2716). The yellow circle represents the search gene *SAUR20*, and the polygons are genes co-expressed with SAUR20. The pink and blue lines indicate positive and negative co-expression relationship with *SAUR20*, respectively. **(B)** Heatmap analysis of all *SAUR* genes in the network of *SAUR20*. The redder the color, the higher the activity of the gene; the bluer the color, the lower the activity of the gene.

Small auxin up RNAs (SAURs), the largest family of early auxin response genes, are involved in the regulation of a wide range of cellular, physiological, and developmental processes (McClure and Guilfoyle, 1987; Ren and Gray, 2015). In RoseAP, evm.model.Chr7.2716 was annotated as *SAUR20*, and the top 300 genes co-expressed with this gene were screened based on the rank of PCC value, with PCC ≥ 0.69. It was found that 28 genes belonged to SAUR-like auxin-responsive protein family, including 11 *SAUR14*, 10 *SAUR20*, 2 *SAUR6*, 1 *SAUR1*, 1 *SAUR23*, 1 *SAUR24*, 1 *SAUR27*, and 1 *SAUR50* (**Figure 4A**). We analyzed the expression profile of these genes and found that these genes showed obvious tissue-specific expression, and were mainly expressed in petals (**Figure 4B**). Furthermore, 23 genes in the *SAUR20*’s network in *R. rugosa* shared orthologous relationship with the *SAUR20’s* network in *A. thaliana.* Nine genes in *R. rugosa* and 6 genes in *A. thaliana* belong to the *SAUR* family, indicating that the *SAUR20* co-expression network was conserved between species, based on a comparison of the top 300 positively co-expressed genes for *SAUR20* among the two species (**Supplementary Figure 3**).

In addition, these genes are simple in structure and short in length, with most of them ranging between 250 bp and 300 bp. Except for five *SAURs* (evm.model.Chr2.4296, evm.model.Chr5.2574, evm.model.Chr5.2575, evm.model.Chr7.2731, and evm.model.Chr7.2757) containing two exons and one intron, the other genes carried only a single exon and no intron; however, all of them contained the Auxin_inducible domain (**Supplementary Table 3**).

Gene duplication is a major characteristic of *SAURs* in plants, including *R. rugosa* (Wu et al., 2012; Chen et al., 2014; Wang et al., 2020). These *SAUR* genes were distributed in clusters, mostly on chromosomes 7 and 6, including nine *SAUR14*, five *SAUR20*, one *SAUR24*, and one *SAUR27* clustered in the reverse strand of chromosome 7. In chromosome 6, *SAUR* genes were located in the forward strand distributed in series except *SAUR50* (evm.model.Chr6.1399). *SAUR6* (evm.model.Chr2.4295) and *SAUR20* (evm.model.Chr2.4296) were distributed in series on the forward strand of chromosome 2 (**Supplementary Table 3**).

### 4.3 Network analysis of dihydroflavonol 4-reductase

**Figure 5:**
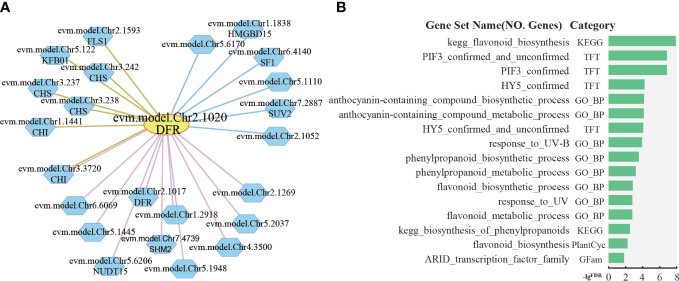
Network analysis of dihydroflavonol 4-reductase. **(A)** The network of *DFR* (evm.model.Chr2.1020). The yellow circle is the search gene *DFR*, and the polygons represent genes co-expressed or protein–protein interactions with *DFR*. The pink, blue, and brown lines indicate positive co-expression, negative co-expression, and protein–protein interaction with *DFR*, respectively. **(B)** Gene set enrichment analysis of all genes in the network of *DFR* by PlantGSAD (http://systemsbiology.cau.edu.cn/PlantGSEAv2/).

The petals of *R. rugosa* are rich in anthocyanins with antioxidant and anti-aging effects. The anthocyanins are synthesized via the flavonoid branch of phenylpropane metabolic pathway and have been widely studied (Kubasek et al., 1992; Winkel-Shirley, 2001; Li et al., 2018). *DFR* gene, which encodes dihydroflavonol 4-reductase that catalyzes the production of leucoanthocyanidin from dihydroflavonols, is an important gene expressed in anthocyanin synthesis (Holton and Cornish, 1995). In *R. rugosa*, evm.model.Chr2.1020 was annotated as *DFR* gene. Further analysis based on co-expression and protein–protein interaction network revealed 11 positive co-expressed genes with edge in pink, 6 negative co-expressed genes with edge in blue, and 7 protein–protein interaction genes with edge in brown (**Figure 5A**). Some of the genes in the network are involved in anthocyanin synthesis, such as Chalcone Synthase (*CHS*), Chalcone Isomerase (*CHI*), Dihydroflavonol 4-Reductase (*DFR*), Flavonol Synthase 1 (*FLS1*), and Kelch domain-containing F-box 1 (*KFB01*). The *CHS* gene encodes chalcone synthase, a rate-limiting enzyme in anthocyanin synthesis (Ferrer et al., 1999). *CHI* gene catalyzes the intramolecular cyclization of bicyclic chalcones into tricyclic (S)-flavanones (Guo et al., 2015), both in the co-expression network and in the protein–protein interaction network of *DFR*. *FLS1* encodes a flavonol synthase that catalyzes the formation of flavonols from dihydroflavonols (Nguyen et al., 2016). KFB protein mediates CHS ubiquitination and degradation (Zhang et al., 2017).

Furthermore, gene set enrichment analysis was performed for those genes in PlantGSAD (http://systemsbiology.cau.edu.cn/PlantGSEAv2/) (Ma et al., 2021), including KEGG, PlantCyc, GO, TFT, and Gfam gene sets. The results showed that some terms related to anthocyanin metabolism were enriched, for example, biosynthesis of phenylpropanoids, flavonoid biosynthesis, and anthocyanin-containing compound biosynthetic process (**Figure 5B**). In addition, we also found two terms related to transcription factors, such as PIF3 confirmed, HY5 confirmed, and response_to_UV-B (**Figure 5B**). HY5, a photomorphogenetic promoter, and PIF3 simultaneously bind to the promoter of anthocyanin biosynthesis gene during the positive regulation of anthocyanin accumulation in plants under light stimulation (Ang et al., 1998; Shin et al., 2007; An et al., 2017).

## 5 Discussion

The development of sequencing technology has led to the availability of a high-quality genome map of *R. rugosa.* However, the integrated analysis of omics data is relatively weak, and the function of a large number of genes is unknown. Therefore, in this study, RoseAP, an online platform for gene function of *R. rugosa*, was built to improve the gene annotation rate.

First, 38,815 genes, covering 97.76% of the coding genes, were annotated functionally and structurally using a variety of algorithms and rules, including Pfam domain, GO, InterPro, trEMBL, SwissProt, KEGG, and analysis of orthologues in 11 species and 8 gene families. Using a case study, the sequence, structure, and function of evm.model.Chr1.1098 gene were determined by ID query. Second, a co-expression network containing approximately 29,657 positive or negative gene pairs, covering 74.7% of the coding genes, was constructed using PCC and MR algorithms. Simultaneously, 1,280 functional modules were mined and PPI networks were constructed. Genes in networks or modules were analyzed via orthologue annotation, gene set enrichment, and expression profiling. Network analysis revealed that the function of genes in the *DFR* network was closely related to anthocyanin metabolism. Several *SAUR* genes of *R. rugosa* shared similar expression patterns.

The SAURs are the largest family of early auxin response genes with ~60 to 140 members in most higher plant species (McClure and Guilfoyle, 1987; Stortenbeker and Bemer, 2018). Similar to other species, SAURs in *R. rugosa* also showed distinct characteristics, such as cluster distribution, simple structure, and tissue-specific expression. A large SAUR cluster was found at position 22,873,890–23,293,079 on chromosome 7 of *R*. *rugosa*, containing 21 SAUR genes (**Supplementary Figure 4**), of which 17 showing similar expression patterns were included in the co-expression network of evm.model.Chr7.2716 (**Figure 4B**). Furthermore, we found that nine of them shared orthologues with SAUR20 co-expression genes in *A*. *thaliana*, indicating that the expression pattern of SAURs was also partially conserved between species (**Supplementary Figure 3**). However, there are still many unknown areas, such as their tissue-specific regulation and evolutionary advantages due to their high levels of cluster retention during the evolutionary process for plant growth adaptation.

We will continue to update the RoseAP database as new data emerge. Since the flower is the main tissue contributing to the economic value of *R. rugosa*, we sequenced petals, pistils, stamens, and the whole flower at the whole genome transcriptome level. The findings comprehensively reflected the gene expression pattern in the flower. A gene co-expression network was thus developed to screen genes with similar expression patterns in the flower. However, this gene co-expression network for *R. rugosa* is still limited by spatiotemporal gene expression. In order to more comprehensively correlate the expression of genes in *R. rugosa*, various factors should be considered during sampling, such as different varieties, tissues, developmental stages, and environmental stress. Nonetheless, the findings are not only limited to the transcriptome, but also defined by the multi-dimensional omics. With the development of sequencing technology, biological characteristics can be presented from different perspectives such as epigenome, proteome, metabolome, phenome, and SNPs to accurately reflect the biological phenomena and internal regulation, thereby contributing to genetic improvement of *R. rugosa*. However, a single species, *R. rugosa*, may not be representative of other plant species. With the gradual accumulation of omics data for other roses, we will upgrade RoseAP into a comprehensive multi-species functional omics analytical platform.

In conclusion, a platform for the functional analysis of *R. rugosa* was constructed based on genomic and transcriptomic datasets. Users can obtain the sequence, structure, and function annotation information based on gene ID for a preliminary analysis of genes. Further analyses of expression profiles, co-expression networks, and PPI networks were provided to explore gene transcriptional activity and functional regulation. In addition, several auxiliary analytical tools, including BLAST, gene set enrichment, orthologue conversion, gene sequence extraction, gene expression value extraction, and JBrowse are currently available. We will continue to update RoseAP to facilitate studies for the genetic improvement of *R. rugosa*.

## Data availability statement

The datasets presented in this study can be found in online repositories. The names of the repository/repositories and accession number(s) can be found in the article/**Supplementary Material**.

## Author contributions

LD performed sample collection, data collection, data analysis, and web server construction. JY helped to construct the web server. JL and HZ helped to prepare the manuscripts. FZ supported server maintenance and database administration. FZ, HL, PS, SS, and XZ helped with sample collection. HZ supervised the project. All authors contributed to the article and approved the submitted version.
