# Supplementary material for: RoseAP: an analytical platform for gene function of Rosa rugosa
Source: Front Plant Sci. 2023 Jun 30;14:1197119. doi: 10.3389/fpls.2023.1197119 (PMC10348015; doi:10.3389/fpls.2023.1197119)
Supplement: Supplementary file 1 [file DataSheet_1.pdf]

Supplementary Material

RoseAP: an analytical platform for gene function of *Rosa rugosa*

Lingling Da[1#], Jiande Li[1#], Fan Zhao[1], Huilin Liu[1], Pengxia Shi[1], Shaoming Shi[1], Xinxin Zhang[1], Jiaotong Yang[2]*, Hui Zhang[1]*

[1]College of Life Science, Northwest Normal University, Lanzhou, China

[2]Resource Institute for Chinese and Ethnic Materia Medica, Guizhou University of Traditional Chinese Medicine, Guiyang, China

# These authors contributed equally to this work.

* Correspondence:

Hui Zhang
zhanghui@nwnu.edu.cn

Jiaotang Yang
y_jiaotong@163.com

1.1 Supplementary Figures

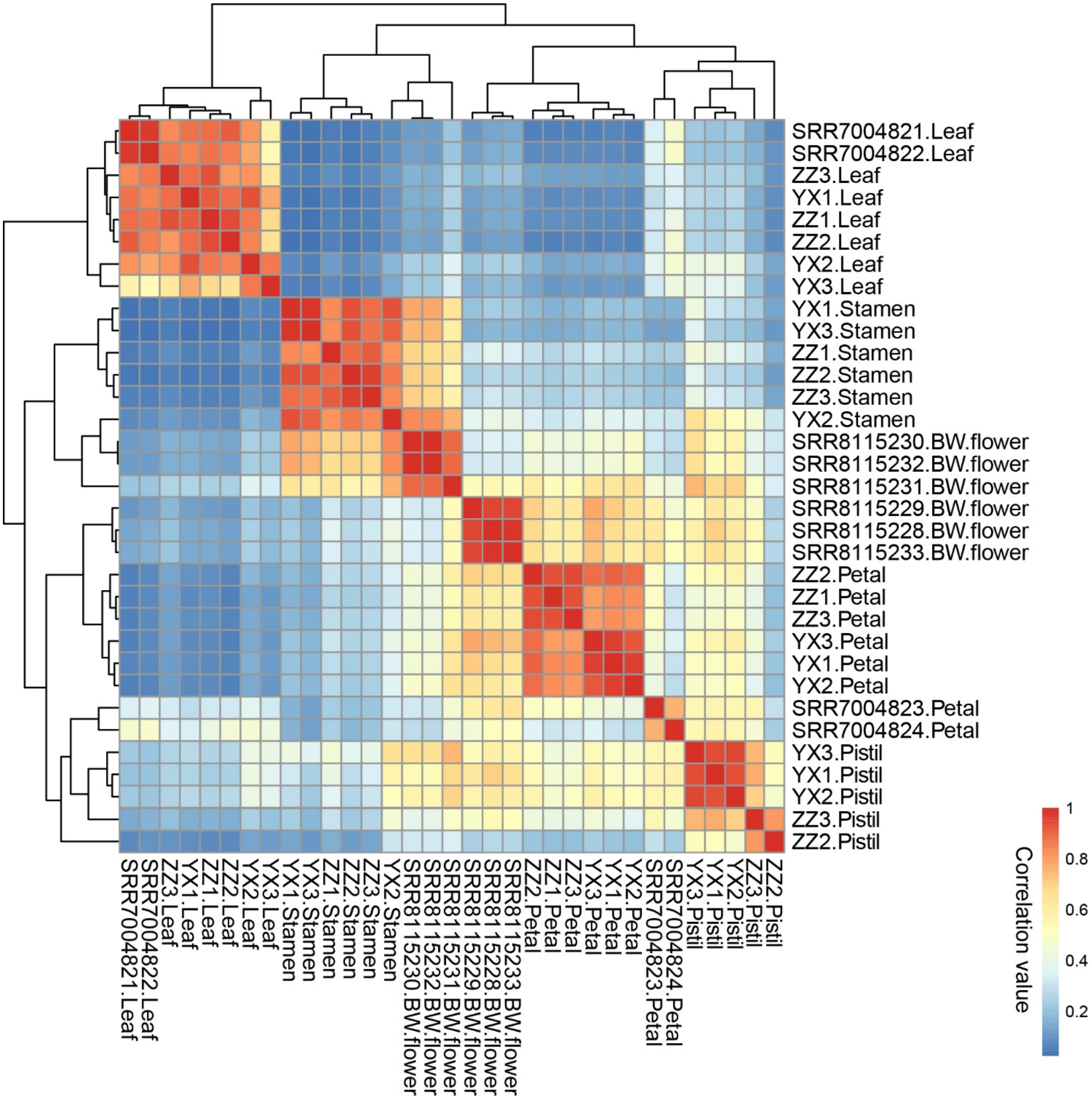

Supplementary Figure 1. Cluster diagram of all RNA-seq data based on TPM value. The color represents sample correlation. The redder the color, the stronger the correlation; the bluer the color, the weaker the correlation.

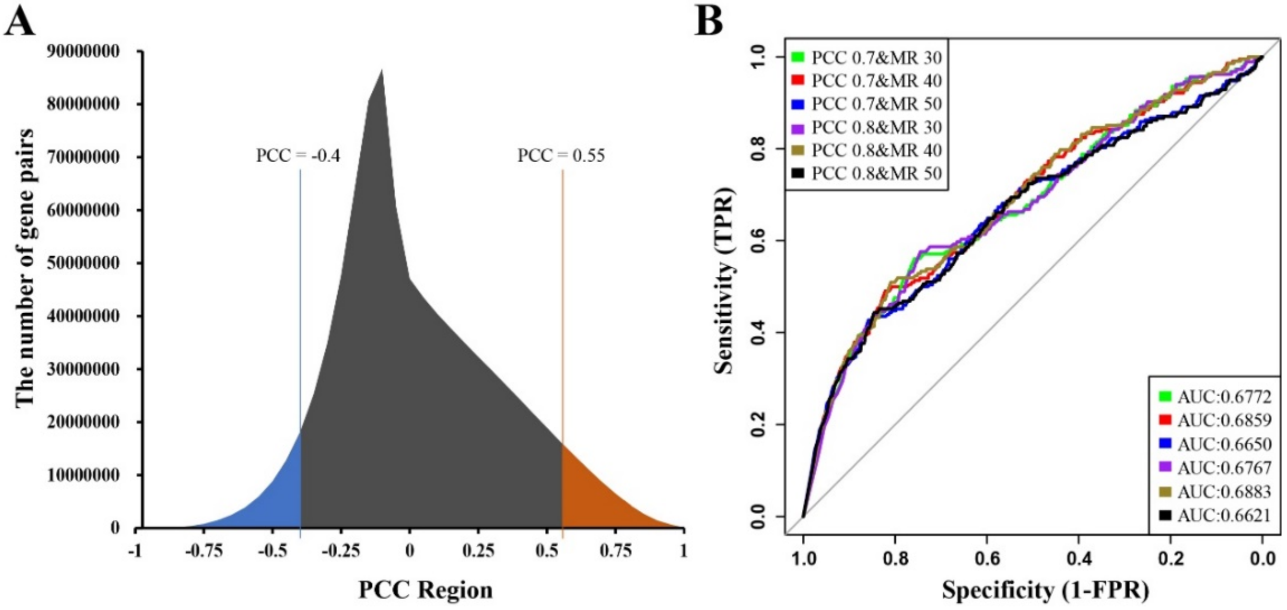

Supplementary Figure 2. Selection of the co-expression network threshold. (A) The distribution of PCC values for all gene pairs. Five percent of the negative co-expressed gene pairs were screened with PCC ⩽ -0.4, and 5% of the positive co-expressed gene pairs were screened with PCC ⩾ 0.55. (B) The AUC value of the co-expression network under different PCC and MR values. A plot of the true-positive rate [TP/TP+FN] versus the false-positive rate [TN/FP+TN] of the positive co-expression network with different PCC and MR values (PCC ⩾ 0.7 & MR ⩽ 30, PCC ⩾ 0.7 & MR ⩽ 40, PCC ⩾ 0.7 & MR ⩽ 50, PCC ⩾ 0.8 & MR ⩽ 30, PCC ⩾ 0.8 & MR ⩽ 40 and PCC ⩾ 0.8 & MR ⩽ 50).

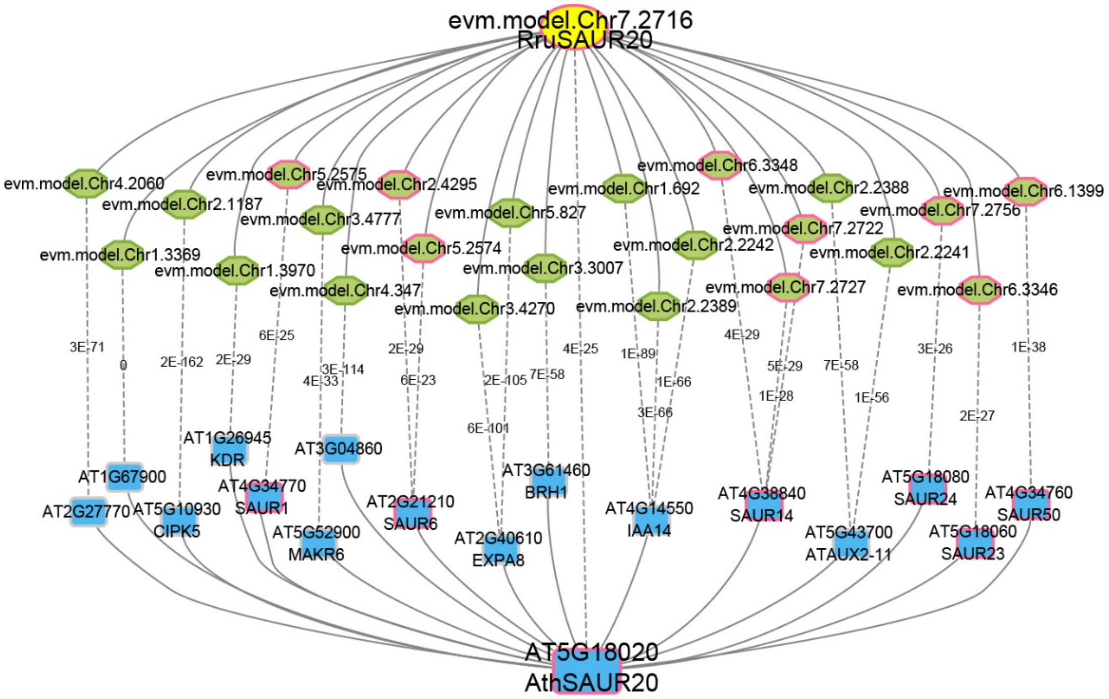

Supplementary Figure 3. Comparison of the top 300 positive co-expressed genes associated with *SAUR20* between *Rosa rugosa* and *Arabidopsis thaliana*. Yellow circle represents evm.model.Chr1.1098 (*RruSAUR20*) of *Rosa rugosa*; green polygons represent genes co-expressed with *RruSAUR20*; blue squares represent genes co-expressed with *AthSAUR20* of *Arabidopsis thaliana*; and SAUR family genes are indicated by red borders. The dashed lines in the middle suggest an orthologous relationship between genes, and the number above is E-value.

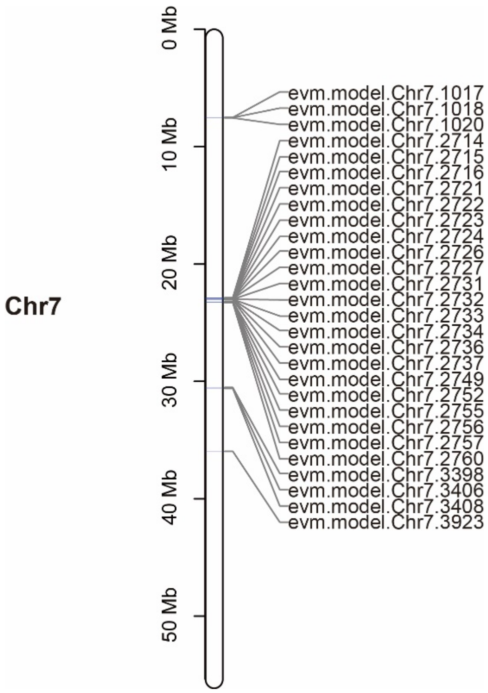

Supplementary Figure 4. Distribution of SAURs on chromosome 7 of *Rosa rugosa*.
